# Caloric restriction promotes functional changes involving short-chain fatty acid biosynthesis in the rat gut microbiota

**DOI:** 10.1038/s41598-018-33100-y

**Published:** 2018-10-03

**Authors:** Alessandro Tanca, Marcello Abbondio, Antonio Palomba, Cristina Fraumene, Fabio Marongiu, Monica Serra, Daniela Pagnozzi, Ezio Laconi, Sergio Uzzau

**Affiliations:** 1 Porto Conte Ricerche, Science and Technology Park of Sardinia, Tramariglio, Alghero Italy; 2 Department of Biomedical Sciences, University of Sassari, Sassari, Italy; 3 Department of Biomedical Sciences, University of Cagliari, Cagliari, Italy

## Abstract

Caloric restriction (CR) is known to promote health and longevity, likely via modification of the gut microbiota (GM). However, functional and metabolic changes induced in the GM during CR are still unidentified. Here, we investigated the short- and long-term effects of CR on the rat GM using a metaproteogenomic approach. We show that a switch from *ad libitum* (AL) low fat diet to CR in young rats is able to induce rapid and deep changes in their GM metaproteomic profile, related to a reduction of the Firmicutes/Bacteroidetes ratio and an expansion of lactobacilli. Specifically, we observed a significant change in the expression of the microbial enzymes responsible for short-chain fatty acid biosynthesis, with CR boosting propionogenesis and limiting butyrogenesis and acetogenesis. Furthermore, these CR-induced effects were maintained up to adulthood and started to be reversed after a short-term diet change. We also found that CR alters the abundance of an array of host proteins released in stool, mainly related to epithelial barrier integrity and inflammation. Hence, our results provide thorough information about CR-induced modifications to GM and host functional activity, and might constitute the basis for novel GM-based approaches aimed at monitoring the effectiveness of dietary interventions.

## Introduction

The gut microbiota (GM) largely derives nutrients from dietary intake. The most densely populated area of the intestine is the distal colon, where the major metabolic activity of bacteria is the fermentation of indigestible oligosaccharides and of carbohydrates that escaped digestion in the small intestine^1^. In addition, a variable amount of proteins, fats and simple sugars also escape small intestine, representing minor dietary substrates for the colonic microbiota. However, a large variability of dietary patterns exists among individuals and, therefore, the diverse quality and quantity of dietary intake might differently feed and modulate the GM, affecting both the microbial composition and the microbial metabolite profile^2–5^.

In this respect, a large number of studies have been reported on the variations of the GM composition occurring according to different diets. The majority of these studies have focused on the comparison of low vs. high energy density (i.e., high fat or high sugar) diets in animals fed *ad libitum* (AL), showing an increase in the Firmicutes/Bacteroidetes ratio and the proliferation of pro-inflammatory Proteobacteria in the latter condition^6–8^.These changes occur rapidly and can be partially restored by reverting to the control diet^9^. Animal experimental data also agree with observational studies in humans, where similar taxonomic features were found to be changed between obese and lean individuals^10,11^.

In addition, the GM composition varies rapidly and significantly in response to macronutrient changes, even when equal numbers of calories are provided^12,13^. This clearly suggests that the relative abundance of the specific GM members strongly depends on the quality of nutrients they have access to. Hence, given the strong relationship among diet, GM and health, there is a growing interest in developing novel dietary strategies to modulate the composition and, possibly, the metabolic functions of the GM.

Among dietary interventions, caloric restriction (CR) is well known for the health-promoting impact on lipid metabolism and longevity^14^. CR is generally applied without changing the macronutrient composition and solely reducing the caloric intake compared to the AL condition. As a consequence, in experimental models, caged individuals fed a CR diet consume completely their food and then fast for several hours before the next feed administration. We have recently reported that CR induces a rapid change (as early as after 3 weeks of CR) of the GM composition in young rats, that parallels a reduction of triglycerides and cholesterol levels in the blood, and that these changes are maintained up to mid age^15^. In particular, a CR diet enabled the expansion of *Lactobacillus* spp. rapidly and persistently up to adulthood^15,16^. CR-induced variation of the GM composition might then play a role in helping extend lifespan and delay the onset of age-related disorders by preserving gut homeostasis. However, the precise biochemical changes the GM undergoes during CR are still undetermined, in the short and in the long term. Of note, combining long-term CR with a high/low fat diet, Wu *et al*. recently found that long-term reduced caloric intake is able to induce metabolic profile changes in urine, including some GM-related metabolites (hippurate, indoxylsulfate, and p-hydroxyphenylacetate), irrespectively of a high- or low-fat diet^17^. Yet, no studies have reported a direct analysis of the protein functions of the microbiota (including enzymes belonging to the main biosynthetic and catabolic pathways) in CR-fed animals.

Here, we aimed to investigate the effect of CR on functions expressed by the GM in a rat model. In a first experiment, we evaluated the active functions differentially displayed in the GM of young rats (fed CR or AL for up to 8 weeks) by using a shotgun metaproteomic approach. In a second experiment, 16S rRNA gene sequencing and metaproteomics were applied to investigate the taxonomic and functional changes induced by long-term CR (1.5 years), and to verify the effects of a short-term reversion to the AL diet. Moreover, taxon-specific functions and metabolic pathways actively working in CR- and AL-fed rat GMs are described and discussed. Finally, we also analysed the relative abundance of host proteins in the faecal samples of the two experimental groups, providing insights on CR/AL-dependent effects on the gut mucosa homeostasis.

## Results

### Caloric restriction induces rapid and deep changes in the faecal microbiota metaproteome

We recently reported that significant compositional changes arise in the faecal microbiota of young growing rats after short-term administration of a CR diet^15^. Here, we sought to verify if those structural changes in the GM are associated with modification in its functional and metabolic profile, through the application of a shotgun metaproteomic approach. To this end, we collected faecal samples from rats undergoing 3, 5 and 8 weeks of CR, as well as from control AL rats at the same time points, and characterised the metaproteomic profile of their GM. A total of 142,942 mass spectra could be matched to 878 different protein functions, belonging to over 250 different microbial genera (complete metrics, identification and annotation data can be found in Supplementary Dataset S1).

**Figure 1:**
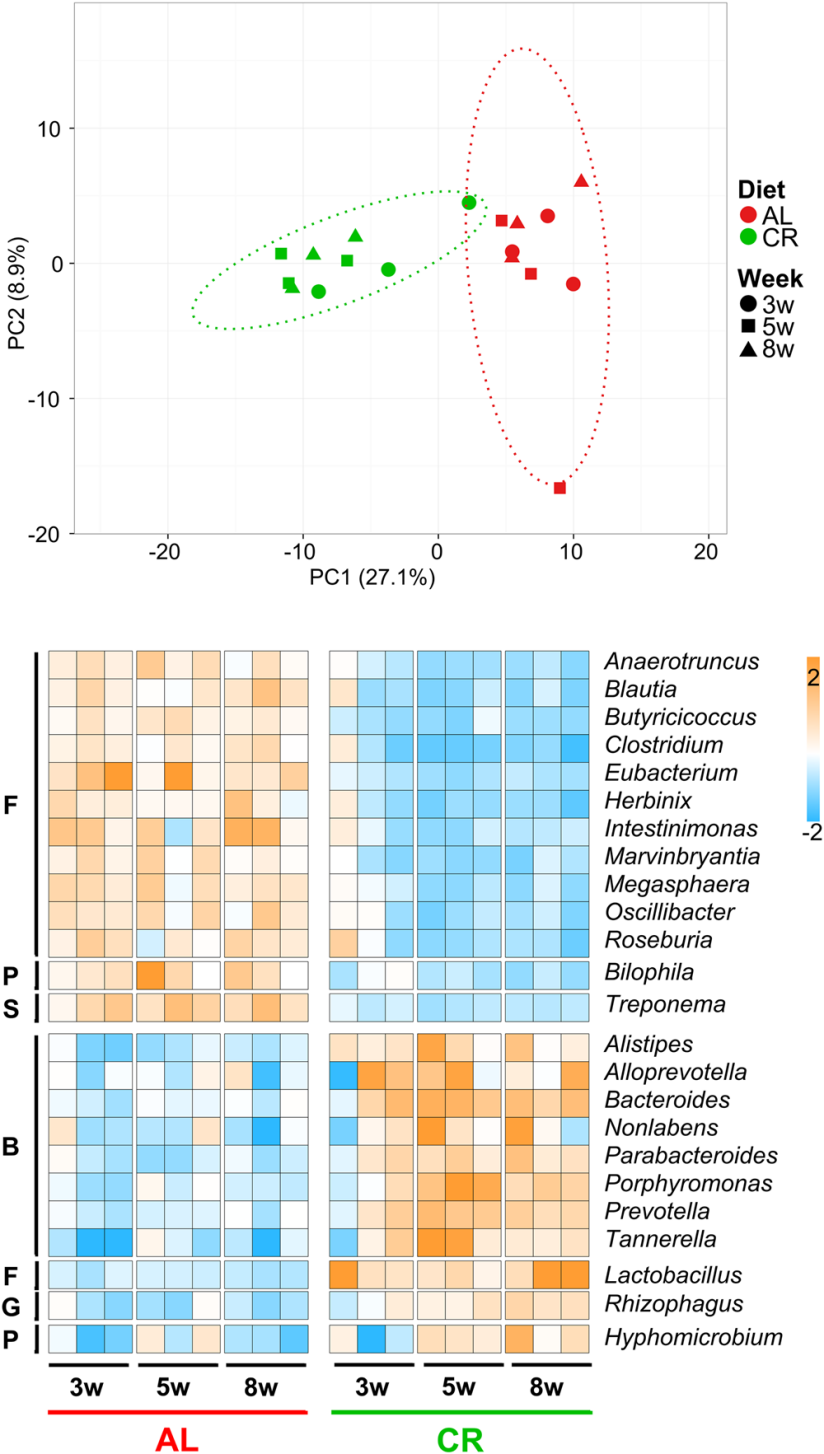
Changes in GM taxonomic composition at genus level, based on metaproteomic results obtained upon caloric restriction treatment on young rats. Top, PCA plot based on microbial genera relative abundance data. Each dot indicates a sample (different time points, expressed in weeks, are illustrated with different shapes), while dotted ellipses indicate 95% confidence level. AL, *ad libitum*; CR, caloric restriction. Bottom, heatmap illustrating microbial genera with significantly differential abundance between AL and CR groups (edgeR analysis followed by Benjamini-Hochberg correction). Columns represent samples, while rows represent genera. The color gradient is based on the standardized abundance (z-score). Only genera with abundance >0.25% are shown, and ordered first according to the group in which they are significantly more abundant, and then based on the phylum to which they belong (B, Bacteroidetes; F, Firmicutes; G, Glomeromycota; P, Proteobacteria; S, Spirochaetes).

First, in order to investigate compositional changes in the GM, we focused on taxonomic data based on the abundance of the proteins expressed by each microbial member. Genus abundance data were initially subjected to a Principal Component Analysis (PCA), revealing that a separation between CR- and AL-fed rats can be observed since 3 weeks of treatment, becoming even clearer up to 8 weeks (p < 0.001 PERMANOVA between groups considering all weeks), as shown in Fig. 1, top. We then performed a differential abundance analysis (edgeR comparison of all AL samples vs all CR samples, followed by Benjamini-Hochberg correction) to identify which genera were mainly responsible for the segregation between groups. Heatmap in Fig. 1 illustrates 24 genera with significantly differential distribution between the GMs of AL- and CR-fed rats. Interestingly, the abundance of 8 Bacteroidetes genera, including *Prevotella* and *Bacteroides*, resulted higher in CR-fed rats compared to AL controls, while 11 Firmicutes genera, including *Clostridium*, *Eubacterium* and *Oscillibacter*, were more abundant in AL compared to CR-fed rats. The only Firmicutes genus to be enriched in CR-fed rats was *Lactobacillus*, with remarkable significance values (FDR = 9 × 10^−20^) and in line with our previous 16S rDNA gene sequencing results^15^.

**Figure 2:**
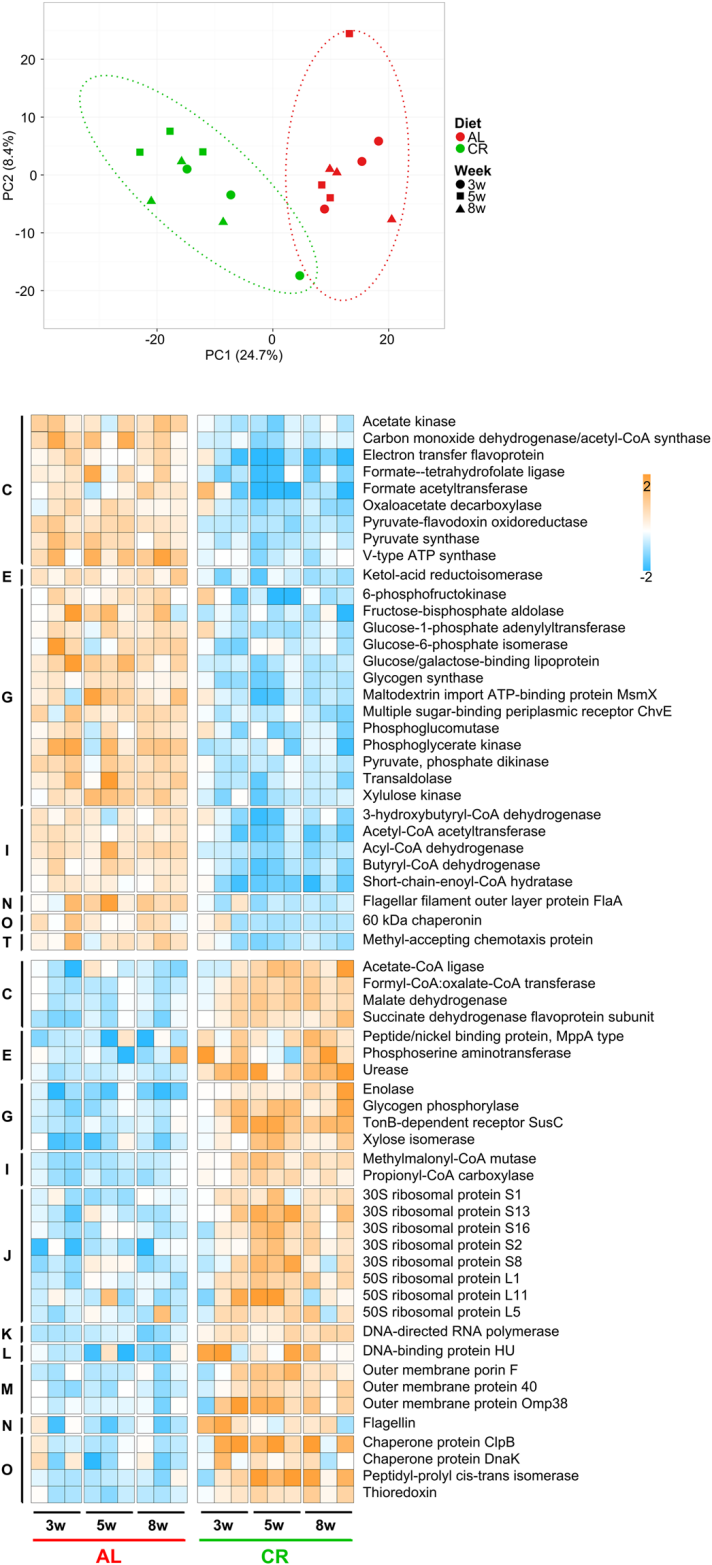
Changes in metaproteome functional expression observed upon caloric restriction treatment on young rats. Top, PCA plot based on relative abundance of microbial protein functions. Each dot indicates a sample (different time points are illustrated with different shapes), while dotted ellipses indicate 95% confidence level. AL, *ad libitum*; CR, caloric restriction. Bottom, heatmap illustrating microbial functions with significantly differential abundance between AL and CR groups (edgeR analysis followed by Benjamini-Hochberg correction). Columns represent samples, while rows represent functions. The color gradient is based on the standardized abundance (z-score). Only functions with abundance >0.25% are shown, and ordered first according to the group in which they are significantly more abundant, and then based on the COG category to which they belong (C, Energy production and conversion; E, Amino acid transport and metabolism; G, Carbohydrate transport and metabolism; I, Lipid metabolism; J, Translation, ribosomal structure and biogenesis; K, Transcription; L, Replication, recombination and repair; M, Cell wall/membrane/envelop biogenesis; N, Cell motility; O, Posttranslational modification, protein turnover, chaperones; T, Signal transduction mechanisms).

Our main interest was directed to elucidate which functional traits could discriminate the activity of the GM of CR- and AL-fed animals. Again, the PCA plot (Fig. 2, top) clearly shows as CR and AL groups can be discriminated based on functional abundance data (p < 0.001 PERMANOVA between groups considering all weeks). Differential abundance analysis (edgeR comparison of all AL samples vs all CR samples, followed by Benjamini-Hochberg correction) allowed us to identify 167 functions that vary significantly between the two groups (the 62 functions exceeding 0.25% of relative abundance in at least one group are listed in the heatmap of Fig. 2). The CR treatment was found to induce a deep rearrangement of the microbial metaproteome, involving both catalytic and structural/antigenic functions belonging to several different COG categories (with “carbohydrate metabolism and transport” being the most represented). In particular, several enzymes responsible for carbohydrate degradation and acetate/butyrate biosynthesis were significantly downregulated after CR, while the expression of various ribosomal, outer membrane, DNA-binding and stress-related proteins appeared to be induced by the CR treatment. In most cases, the differential trend started after 3 weeks of treatment and reached similar, top values at 5 and 8 weeks.

### Caloric restriction-induced metaproteomic changes in the faecal microbiota are kept in the adulthood but start to be reversed after 1 week of *ad libitum* diet

We recently demonstrated that compositional changes early produced by CR in the rat faecal microbiota mainly involve *Lactobacillus* spp. and are maintained up to 9 months of dietary intervention^15^. Here, we aimed to investigate, under a structural and functional perspective: (i) if these changes can be still observed in older rats; (ii) if reverting from long-term CR diet to short-term AL diet causes a shift of the faecal microbiota composition and activity. To this purpose, we collected faecal samples from rats treated with CR for 1.5 years, as well as from control AL rats at the same time point; then, CR-fed rats were switched back to the AL diet, and their faecal samples were collected after a week (CR → AL). DNA and proteins were extracted from faecal samples, with the aim of carrying out both 16S rRNA gene sequencing and metaproteome analysis. 16S rRNA gene sequencing provided 1,429,463 reads, assigned to 217 different microbial genera (detailed information is provided in Supplementary Dataset S2). When considering metaproteomic analysis, a total of 126,913 mass spectra could be matched to 1,000 different protein functions, belonging to 241 different microbial genera (complete metrics, identification and annotation data can be found in Supplementary Dataset S1).

**Figure 3:**
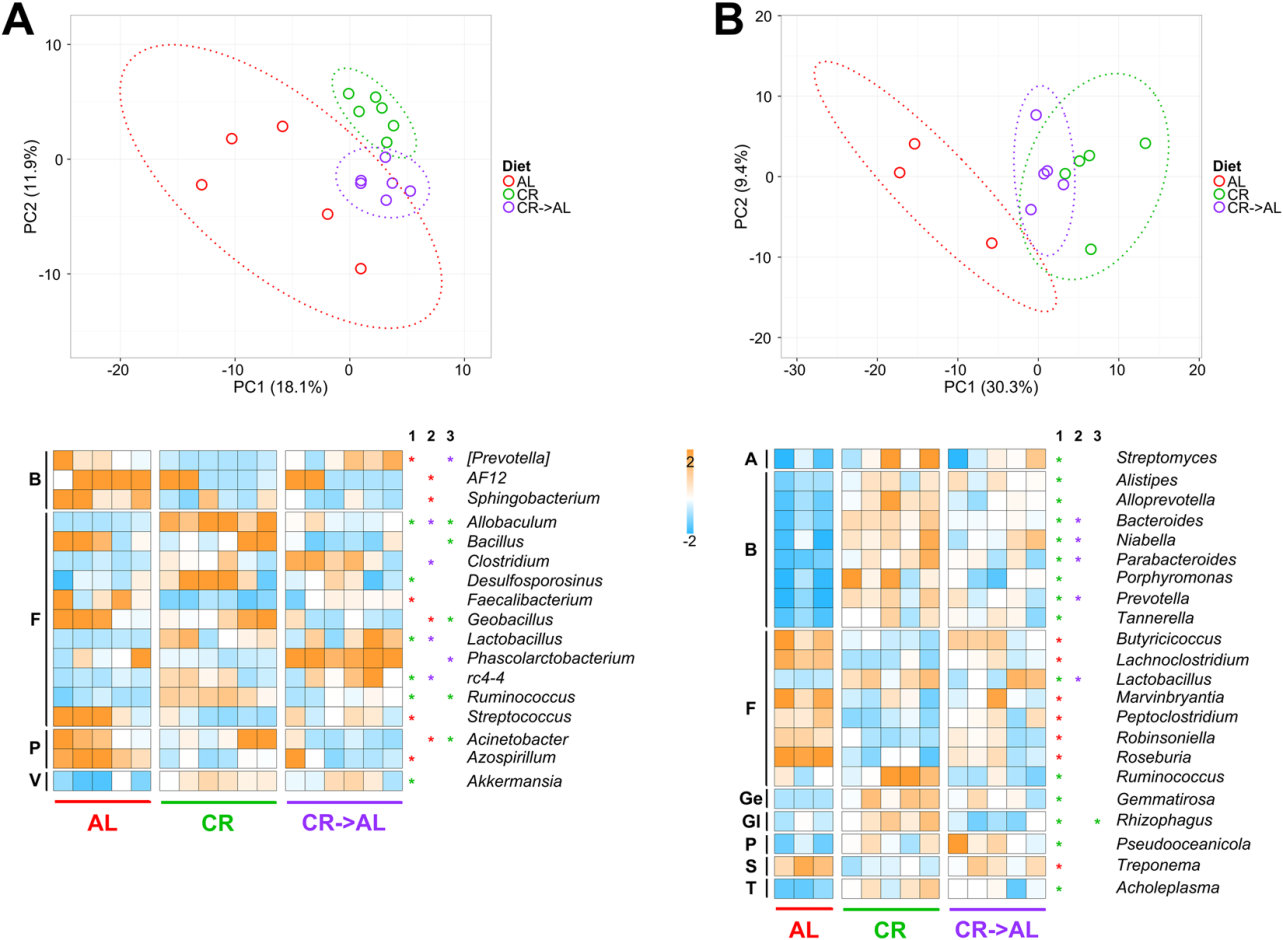
Changes in taxonomic composition at genus level based on 16S rDNA gene sequencing (**A**) and metaproteomics (**B**) results obtained on adult rats (1.5 years of treatment). AL, *ad libitum*; CR, caloric restriction; CR → AL, 1-week reversion from caloric restriction to *ad libitum*. (**A**) Top, PCA plot based on 16S rDNA gene sequencing relative abundance data at the genus level. Each dot indicates a sample, while dotted ellipses indicate 95% confidence level. Bottom, heatmap illustrating microbial genera with significantly differential abundance between groups upon edgeR analysis followed by Benjamini-Hochberg correction. Heatmap columns represent samples, while rows represent genera. Asterisks in supplementary columns 1, 2 and 3 indicate genera with significantly differential abundance upon AL *vs* CR, AL *vs* CR → AL, and CR *vs* CR → AL comparisons, respectively. The asterisk colour refers to the group in which the genus was found as more abundant. The color gradient is based on the standardized abundance (z-score). Only genera with abundance >0.1% are shown, and ordered according to the phylum to which they belong (B, Bacteroidetes; F, Firmicutes; P, Proteobacteria; V, Verrucomicrobia). (**B**) Same as in (**A**), but concerning metaproteomic data instead of 16S rDNA gene sequencing data. Further phylum abbreviations: A, Actinobacteria; Ge, Gemmatimonadetes; Gl, Glomeromycota; S, Spirochaetes; T, Tenericutes.

Figure 3 shows taxonomic results from 16S rDNA (A) and metaproteomic (B) analyses. Long-term CR was found to induce structural changes that are kept up to 1.5 years of dietary treatment, as clearly illustrated by PCA plots (top) based on relative genus abundances. Even more interestingly, samples collected from rats after only one week of “diet reversion” (CR → AL) clustered in between AL and CR groups, indicating a perturbation of the GM composition in these aged animals due to an increase in the administered quantity of the very same feed. Comparing the three groups analysed, according to 16S rDNA and metaproteomic data, a number of microbial genera showed a significantly differential abundance (again, edgeR comparison of all AL samples vs all CR samples, followed by Benjamini-Hochberg correction), as depicted in the heatmaps of Fig. 3. On the whole, genus abundances were quite heterogeneously distributed among groups and among phyla according to 16S rDNA data; conversely, a clearer trend could be observed based on metaproteomic profiles, with CR → AL abundances placing almost always in the middle between AL and CR, and ‒ consistently with the results obtained on younger rats ‒ all Bacteroidetes differential genera being enriched in CR-treated rats. A consensus could be found between 16S rDNA and metaproteomic results when considering *Lactobacillus* and *Ruminococcus* spp., which were found to be more represented in CR microbiota compared to AL controls according to both approaches. The quite low consensus between 16S rDNA and metaproteomic differential results may be mainly due to the use of two different taxonomic databases (Greengenes and NCBI/UniProt, respectively).

**Figure 4:**
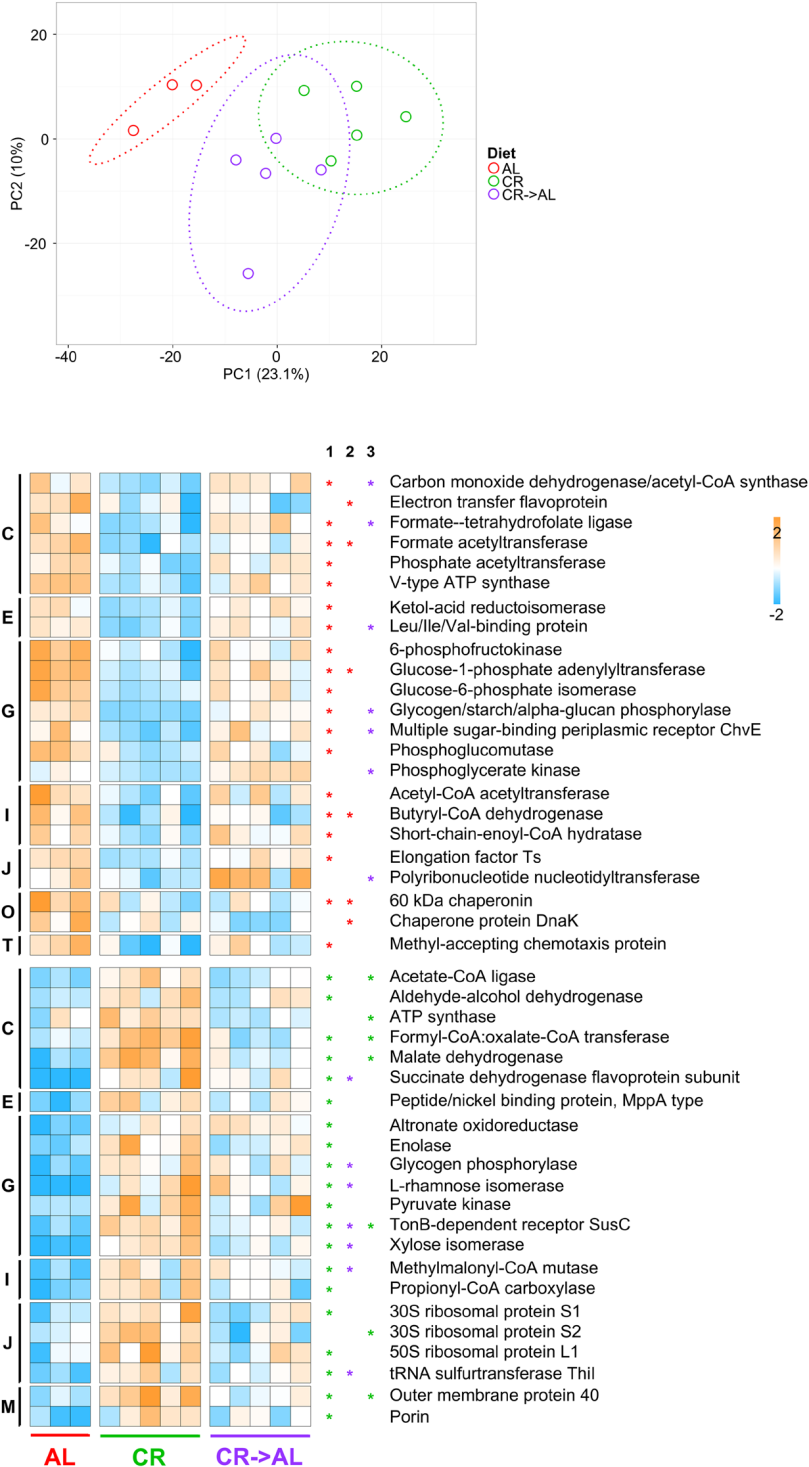
Changes in metaproteome functional expression observed upon caloric restriction treatment on adult rats (1.5 years of treatment). AL, *ad libitum*; CR, caloric restriction; CR → AL, 1-week reversion from caloric restriction to *ad libitum*. Top, PCA plot based on relative abundance of microbial protein functions. Each dot indicates a sample, while dotted ellipses indicate 95% confidence level. Bottom, heatmap illustrating microbial functions with significantly differential abundance between groups upon edgeR analysis followed by Benjamini-Hochberg correction. Heatmap columns represent samples, while rows represent functions. Asterisks in supplementary columns 1, 2 and 3 indicate functions with significantly differential abundance upon AL *vs* CR, AL *vs* CR → AL, and CR *vs* CR → AL comparisons, respectively. The asterisk colour refers to the group in which the function was found as more abundant. The color gradient is based on the standardized abundance (z-score). Only functions with abundance >0.25% are shown, and ordered first according to the group in which they are significantly more abundant, and then based on the COG category to which they belong (C, Energy production and conversion; E, Amino acid transport and metabolism; G, Carbohydrate transport and metabolism; I, Lipid metabolism; J, Translation, ribosomal structure and biogenesis; M, Cell wall/membrane/envelop biogenesis; O, Posttranslational modification, protein turnover, chaperones; T, Signal transduction mechanisms).

The metaproteome dataset was further investigated to identify differences in the expression of protein functions. Once assessed that functional data led to a PCA-based group clustering consistent to that observed for taxonomic data (Fig. 4, top), we focused our attention on differential functions. A total of 113 functions were found with significantly differential abundance among the three sample groups (as a result of edgeR comparison of all AL samples vs all CR samples, followed by Benjamini-Hochberg correction); among them, 73 (65%) had also been consistently found as differential in the young rat experiment. Heatmap in Fig. 4 lists 45 differential functions, exceeding 0.25% of relative abundance in at least one group, belonging to many different COG categories (“carbohydrate metabolism and transport” was again the most represented). Consistent with the data obtained in the young rat experiment, enzymes responsible for acetate/butyrate biosynthesis (carbon monoxide dehydrogenase/acetyl-CoA synthase, formate–tetrahydrofolate ligase and phosphate acetyltransferase for acetogenesis; acetyl-CoA acetyltransferase, butyryl-CoA dehydrogenase and short-chain-enoyl-CoA hydratase for butyrogenesis) were significantly downregulated after CR, while the expression of those involved in propionogenesis (methylmalonyl-CoA mutase and propionyl-CoA carboxylase) appeared to be induced by the CR treatment. Several catalytic functions participating to pentose metabolism (including L-rhamnose and xylose isomerases and altronate oxidoreductase) were also revealed to be consistently more abundant in CR samples. In addition, in almost all cases of differential protein abundance between AL and CR rats, levels in CR → AL rats were in between those measured in CR and AL rats, confirming that a single week of “diet reversion” is sufficient to induce changes in the GM functional profile.

### Genus-specific functional analysis reveals peculiar functional shifts related to caloric restriction

**Figure 5:**
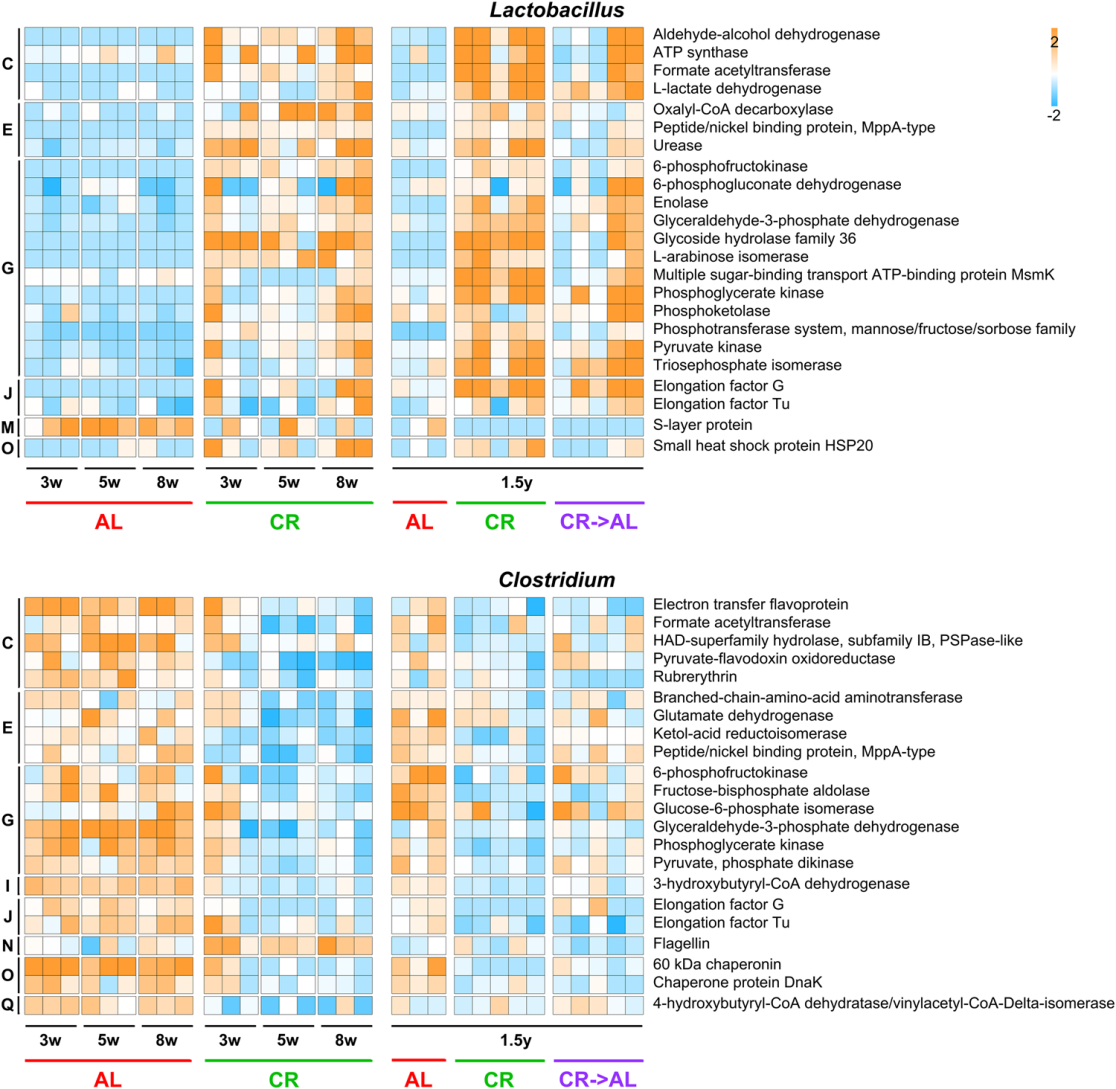
Functional expression profile of *Lactobacillus* (top) and *Clostridium* (bottom) metaproteomes. Relative abundance values concerning the young rat experiment (left, up to 8 weeks of treatment) and adult rat experiment (right, 1.5 years of treatment) are shown. AL, *ad libitum*; CR, caloric restriction; CR → AL, 1-week reversion from caloric restriction to *ad libitum*. Heatmap columns represent samples, while rows represent functions. The color gradient is based on the standardized abundance (z-score). Only functions with abundance >0.1% (*Lactobacillus*) and >0.05% (*Clostridium*) are shown (ribosomal proteins were excluded), and ordered according to the COG category to which they belong (C, Energy production and conversion; E, Amino acid transport and metabolism; G, Carbohydrate transport and metabolism; I, Lipid metabolism; J, Translation, ribosomal structure and biogenesis; M, Cell wall/membrane/envelop biogenesis; N, Cell motility; O, Posttranslational modification, protein turnover, chaperones; Q, Secondary structure).

To gain insight into the contribution of specific microbial genera to the functional activity of the GM in CR- and AL-fed rats, we combined functional and taxonomic (genus level) annotations and investigated the abundance profile of the main proteins expressed by some of the most represented members of the faecal microbiota, including *Lactobacillus*, *Clostridium* (see Fig. 5), *Bacteroides*, *Prevotella*, *Oscillibacter* and *Ruminococcus* (see Supplementary Fig. S1).

*Lactobacillus* was found to be significantly enriched in CR samples at all the time points analysed in this study, consistently with previous reports^15^. Therefore, we were particularly interested in understanding which functions and metabolic activities related to *Lactobacillus* spp. are actually increased in the CR microbiota. Among *Lactobacillus*-specific proteins significantly more abundant in the CR metaproteome, we found many enzymes involved in hexose (including glycolytic enzymes, but also alpha-galactosidases comprised in the glycoside hydrolase family 36), pentose (belonging to pentose phosphate and pentose-glucuronate interconversion pathways) and pyruvate (to lactate and formate) metabolism, but also in oxalate and urea degradation. Proteins responsible for transport and phosphorylation of carbohydrates were also observed as higher in CR-fed rats. Intriguingly, a single *Lactobacillus* protein function followed the opposite trend (significantly higher in AL-fed rats), i.e. the S-layer protein.

*Clostridium* spp. were instead reduced after CR treatment, with a consequent and significant decrease in expression of various clostridial functions, including several enzymes mainly involved in glycolysis, but also in pyruvate and butyrate metabolism. Furthermore, only flagellin, a clostridial protein with relevant antigenic properties, was strikingly observed as higher in CR-fed rats.

When looking at the two main Bacteroidetes genera, namely *Bacteroides* and *Prevotella*, we found several proteins consistently more abundant in the CR rat faecal microbiota, mainly involved in membrane transport, metabolism and protein folding. *Bacteroides* enzymes responsible for pentose catabolism and *Prevotella* proteins belonging to the TonB-dependent transport system were typically increased in the CR metaproteome.

We also examined the metaproteome of two Firmicutes members with different behaviour, *Oscillibacter* and *Ruminococcus*. *Oscillibacter* proteins, similarly to most Firmicutes, were depleted in the CR metaproteome, including several enzymes participating to butyrate, pyruvate and acetate metabolic pathways. On the contrary, we observed an enrichment of *Ruminococcus* after long-term CR, mainly related to the overexpression of cellulases (glycoside hydrolase family 9).

### Caloric restriction promotes expression of propionogenic enzymes and limits abundance of butyrogenic and acetogenic enzymes

**Figure 6:**
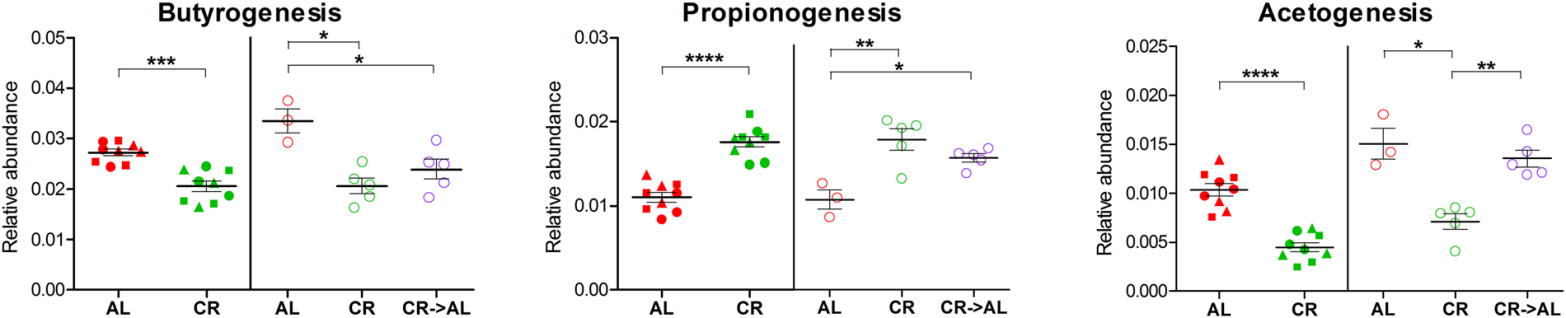
Scatter plots showing the relative abundance of enzymes involved in short-chain fatty acid biosynthesis. AL, *ad libitum*; CR, caloric restriction; CR → AL, 1-week reversion from caloric restriction to *ad libitum*. Each dot indicates a sample; different time points are illustrated with different shapes (same as in Fig. 1). The young rat experiment results are shown on the left of each scatter plot, while the adult rat experiment results are shown on the right. *p < 0.05; **p < 0.01; ***p < 0.001; ****p < 10^−5^.

We noticed that several enzymes implicated in short-chain fatty acid (SCFA) biosynthesis exhibited differential expression between CR and AL faecal microbiota. To go deeper in the characterisation of these relevant metabolic pathways, we inspected the identified functions in search for all catalytic activities related to butyrate, propionate and acetate metabolism, according to the orthologous genes listed in the corresponding KEGG pathways. First, we calculated the global relative abundance of all enzymes contributing to butyrogenesis, propionogenesis and acetogenesis in the different study groups (listed in Supplementary Fig. S2), and found ‒ consistently both in young and adult rats ‒ that propionogenic functions were significantly higher in the CR metaproteome, whereas a decrease in proteins participating to butyrate and acetate biosynthesis was observed in CR-fed rats (Fig. 6). Interestingly, adult rats fed AL for 1 week after 1.5 years of CR treatment showed a rapid restoration of the levels of acetogenic enzymes typical of an AL diet, while changes in butyrogenic and propionogenic enzyme abundances appeared to be slower.

To investigate the taxonomic contribution to these metabolic pathway, a map illustrating the expression profiles of the SCFA biosynthetic enzymes identified in this study, including the related taxonomic classification, was generated (Supplementary Fig. S2). A high level of cross feeding appears to occur for butyrogenesis, as each pathway step was found to be due to different members of Clostridia (including *Butyricicoccus*, *Butyrivibrio*, *Clostridium*, *Eubacterium*, *Faecalibacterium*, *Oscillibacter* and *Roseburia*), while the central, typical reactions of propionate biosynthesis (catalysed by methylmalonyl-CoA mutase and propionyl-CoA carboxylase) could be attributed mainly to *Bacteroides* and *Prevotella* spp. Of note, abundance data revealed that the expression of the acetogenic carbon monoxide dehydrogenase (assigned mostly to *Blautia*) was rapidly and strongly induced in formerly CR-fed rats after just a week of AL feeding, restoring abundance levels comparable to those reached in the long-term AL-fed group.

### Caloric restriction modifies abundance of some relevant host proteins detectable in stool

In addition to the microbial portion of a stool sample (microbiota), metaproteomics allows the characterisation of the host proteins that are released in the gut lumen and embodied within the faecal material. We therefore compared the host proteins abundance profile in AL- and CR-fed rats, both after short-term (3–8 weeks) and long-term (1.5 years) dietary treatment (Supplementary Tables S1 and 2). Of interest, enzymes involved in lipid degradation, including bile salt-activated lipase, neutral ceramidase and inactive pancreatic lipase related protein 1, were detected as more abundant in the CR faecal proteome in young and older rats. Further, host proteins involved in mucus production and mucosal anti-inflammatory response were higher in the gut of AL-fed rats (i.e., murinoglobulin-1, calcium activated chloride channel regulator 1 and mucin-2), together with indicators of cell death burden (cytochrome c and actin). Vice versa, a number of keratins, whose alteration in colon is reported during inflammatory stress^18^, were more abundant in CR-treated rats, including keratin 1, a protein that is key in maintaining epithelial barrier and in the control of intestinal mucosa permeability.

## Discussion

As diet is one of the most relevant factors shaping the GM, dietary interventions and targeted nutritional therapies hold promise for preventing intestinal dysbiosis and promote host health^19^. A growing number of studies have been investigating the impact of specific foods or dietary regimens on GM composition and activity and on the metabolic status of the host^20^. In this context, the effect of quantitative (only) dietary changes, such as CR, has been less explored. Furthermore, most studies aiming at characterising the impact of dietary interventions on the GM are currently performed using 16S rRNA gene sequencing, able to provide a snapshot of the taxonomic structure of the microbial community. However, functional meta-omic approaches, such as metaproteomics, should be used to infer information about the functions and metabolic pathways that are actually active in the GM. Therefore, we decided to apply an established metaproteogenomic pipeline^21^ to gain insights into the functional changes occurring in the GM of young and older rats subjected to CR.

Under a taxonomic perspective, the metaproteomic data presented in this study show a global increase of Bacteroidetes taxa and decrease of Firmicutes taxa (apart from *Lactobacillus*) after CR, compared to the AL control. Longitudinal changes in the Firmicutes/Bacteroidetes (F/B) ratio have been linked for years to obesity (higher F/B ratio) and weight loss (lower F/B ratio)^22,23^. Furthermore, our results are in line with a few previous studies describing the effect of CR in animal models or human individuals. Xu *et al*. examined the GM of C57BL/6N female mice fed various dietary interventions (including CR) and found a higher abundance of Bacteroidetes (as well as *Bacteroides*, at the genus level) in CR-fed mice compared to control or high-fat diets^24^. In another report, Ruiz and coworkers observed a marked reduction of the F/B ratio after one-year of dietary pattern with a lower intake of fat and carbohydrates in obese adolescents^25^. The previously described blooming of *Lactobacillus* spp. after short- and long-term CR in rat^15^ has been clearly confirmed in this study, consistently with other reports^16,24^. Lactobacilli are well-known probiotics able to modulate the immune system and to alter the lipid metabolism, and their abundance has been reported to be inversely associated with inflammation^26^.

The metaproteomic approach used in this study allowed the analysis of the functions actively expressed by the GM, and therefore to link their changes in abundance to the CR treatment. The main evidence we observed is related to SCFA metabolism, as the expression of enzymes involved in propionate biosynthesis (mainly due to members of Bacteroidetes) was found to be induced by CR, whereas those responsible for butyrate and acetate biosynthesis (mainly due to anaerobic Firmicutes) were more abundant in AL-fed rats. SCFA production by the GM has been increasingly investigated over the last years, as SCFAs have been found to have a deep impact on the host-GM metabolic homeostasis^20^. In line with the results presented here, Liou and colleagues found increased levels of propionate associated with the GM of mouse models of gastric bypass, and demonstrated that the GM from gastric bypass-treated mice is able to confer protection from diet-induced obesity upon transfer to germ-free recipient mice^27^. Furthermore, in humans, the delivery of propionate-esterified carbohydrates to the colon led to weight gain reduction in a randomised study of overweight adults^28^. On the other hand, a recent study in rats demonstrated that the increased production of acetate by a dysbiotic GM is able to promote insulin and ghrelin secretion, hyperphagia and obesity^29^. Interestingly, several tumours appears to depend on the availability of acetate as a metabolic substrate^30^, and long-term exposure to CR diet was recently shown to delay the growth of transplanted pre-neoplastic hepatocytes^31^. Butyrate, in turn, is both associated to energy production and weight gain (being a preferred substrate for colonocytes) and to anti-inflammatory and immune system-regulating functions^32,33^. Recently, butyrylated starch fermentation, followed by butyrate release in colon, has also been reported to enable protection against type 1 diabetes by increasing the number and the function of T regulatory cells in the NOD mice model^34^. Hence, CR and other dietary interventions might be exploited to modulate the SCFA production by GM; however, careful attention should be paid when designing microbiome-based approach to control weight gain and metabolism in patients with immunological disorders.

Focusing on genus-specific functions, we found that most proteins exhibiting high expression rate and CR-related variations in abundance were enzymes involved in carbohydrate metabolism, indicating - as expected - that this is a key activity for the GM and it is strongly modulated by diet^35^. Of note, two structural proteins known to interact with the host and other microbes, namely S-layer protein and flagellin from *Lactobacillus* and *Clostridium*, respectively, presented an opposite expression trend compared to the other proteins from the same microbial genus. S-layer proteins are crystalline proteins that line over the bacterial membrane and adhere to the epithelial cell surface, enhancing bacterial-cell adhesion^36^. Their expression increase when bacteria enter the stationary phase^37^. Thus, their abundance in faecal sample of AL-treated rats is in agreement with a reduced growth rate and relative lower abundance of the *Lactobacillus* genus. Further, S-layer proteins were suggested to play a role in the intestinal bacteria-to-bacteria competition (pathogen exclusion) activity of *Lactobacillus* probiotic strains^38,39^. Therefore, S-layer protein abundance in adverse environmental conditions might enable *Lactobacillus* spp. to partially control the over-colonisation by other, co-existing, bacterial groups. Clostridial flagella are known to exert a peculiar role in adhesion, colonisation and pathogenicity^40^. Further, FliC protein has been reported to have a pleiotropic effect on *Clostridium* gene expression, exerting a control over a number of membrane transport systems, as well as over carbon metabolism and sporulation^41^. Thus, the increased expression of *Clostridium* flagellin in the faecal samples of the CR-fed rats is consistent with a situation where *Clostridium* is not occupying its fundamental ecological niche, as hypothesised by Barketi-Klai *et al*.^41^.

We also compared the abundance of host proteins between CR- and AL-fed rats, as faecal metaproteomics allows the simultaneous identification of microbial and host proteins^42,43^. Bile salt-activated lipase, also known as carboxyl ester lipase, was detected at significantly higher abundance in the CR-fed faecal proteome, compared to the AL controls. This enzyme has a key role in the digestion of triglycerides (as well as of fat-soluble vitamin esters and cholesterol esters), and changes in its expression have been described in animal models of obesity associated with high-fat diet^44^. A similar trend was observed also for neutral ceramidase, which is known to be released in the gut lumen where it catalyses hydrolysis of sphingolipids^45,46^. This increase in stool of enzymes involved in lipid degradation is consistent with the well-known impact of CR (and related fasting) on lipid metabolism^47^ and with the increase in number and size of lipid droplets observed the in *Caenorhabditis elegans* intestine after CR^48^. Hence, these data provide new insights on the metabolic state associated with intestinal homoeostasis and aging retardation by long term CR.

On the contrary, murinoglobulin-1, calcium activated chloride channel regulator 1 and mucin-2 were detected in higher proportions in the control AL-fed rats. The former is known to be involved in the mucosal anti-inflammatory response as a protease inhibitor^49^. Accordingly, mucin production is also assessed as a common defence mechanism to protect the underlying mucosa against pathogens, and calcium activated chloride channel regulator 1 may be involved in the regulation of mucus production and/or secretion by goblet cells^50^. Furthermore, actin, among the most abundant host cell proteins, was released in higher amount in faeces of the AL group, possibly due to more intense exfoliation processes. On the other hand, we report here that the gut metaproteome of AL-fed animals show a dramatic and significant reduction of numerous keratins, whose role is well known in maintaining epithelial barrier functions^51^.

The compositional and functional GM shift after only one week of diet reversion (CR → AL) is another salient, although preliminary, finding presented in this work. The existence of a fast response of the GM to diet has been described in human studies, although the GM may be able to counterbalance diet-induced changes due to its resilience properties^20,52^. As the reversion experiment stopped after one week, we could not verify if the CR treatment reversion has a long-lasting impact on the GM; further studies will be needed to elucidate this aspect.

It is important to note that diet regimens (including CR) may have highly variable effects on different subjects/animal models owing to the individualised nature of the GM^20^. Therefore, we cannot rule out that taxonomic and functional changes described in this study might be different when using other animal models (e.g. mouse) and, most importantly, when shifting to human populations (usually displaying a remarkable inter-individual variability in the GM composition). Furthermore, the effects of CR on the GM might change depending on the food composition (here rats were fed a standard, low fat, lab chow). Finally, we acknowledge that state-of-the-art metaproteomics (and meta-omic approaches in general) still suffers from some limitations, especially concerning data analysis and annotation (e.g. a low annotation yield at the lowest taxonomic levels), which are going to be hopefully overcome along with developments in microbiome bioinformatics, the release of more and more complete databases, and the application of refined and standardised analysis protocols.

In conclusion, CR is able to induce rapid and deep taxonomic and functional changes in the rat GM which are maintained up to adulthood. In compositional terms, CR causes a reduction of the F/B ratio along with an expansion of lactobacilli; in functional terms, CR specifically promotes expression of propionogenic enzymes and limits abundance of butyrogenic and acetogenic enzymes. Furthermore, these effects appear to start to be reversed after a short-term diet change. We also found host proteins secreted in stool (including bile salt-activated lipase) whose expression level correlates with the CR treatment. Taken together, these results provide useful and detailed indications about CR-induced GM dynamics, and might constitute the basis for the development of novel approaches aimed at monitoring diet effects and efficacy, as well as of possible future therapeutic applications.

## Methods

### Animals and samples

A total of 23 Fisher 344 rats were used from a colony available at the Department of Biomedical Sciences, University of Cagliari. Animals were fed with Purina Rodent Lab Chow diet with 3% of fat (Mucedola srl, Settimo Milanese, Italy). Animal studies were reviewed and approved by the Institutional Animal Care and Use Committee of the University of Cagliari. Animal experiments were performed in accordance with the relevant guidelines and regulations.

In a first experiment, 12 rats were fed AL until 8 weeks of age; then, 6 of them were randomly selected to form a separate group and were given 70% of the AL ratio (CR). Food to CR group was delivered nightly (at 1AM) through a computer-assisted automated food dispenser. Faecal samples were collected at 3, 5 and 8 weeks after division in two groups.

In a second experiment, 11 rats were fed AL until 15 weeks of age; then, 6 of them were randomly selected to form a separate group (CR) and were fed as described above. Faecal samples were collected at 1.5 years after division in two groups. Then, rats in the CR group were returned to AL feeding (CR → AL); faecal samples were collected 1 week after the dietary change.

All collected faecal samples (N = 53) were immediately stored at −80 °C until use. At the time of the analyses, stool samples were thawed at 4 °C, and, for the second experiment, two portions were collected from each of them for protein and DNA extraction, respectively.

### Protein extraction and metaproteomic analysis

Faecal samples were subjected to bead-beating and heating/freezing steps after resuspension in an SDS-based, reducing extraction buffer, as described earlier^21^. Protein extracts were cleaned up, alkylated and trypsin digested according to the filter-aided sample preparation (FASP) procedure^53^, with minor modifications illustrated elsewhere^43,54^. Peptide mixtures concentration was estimated by measuring absorbance at 280 nm with a NanoDrop 2000 spectrophotometer (Thermo Fisher Scientific, Waltham, MS, USA), using dilutions of the MassPREP *E. coli* Digest Standard (Waters, Milford, MA, USA) to generate a calibration curve. In the first experiment, tryptic digests coming from rats reared in the same cage (2 rats per cage) were equally pooled after quantification.

LC-MS/MS analyses were carried out using an LTQ-Orbitrap Velos mass spectrometer (Thermo Fisher Scientific) interfaced with an UltiMate 3000 RSLCnano LC system (Thermo Fisher Scientific). The single-run 1D LC peptide separation was performed as detailed elsewhere^21^, applying a 247 min separation gradient and loading 4 μg of peptide mixture per sample. The mass spectrometer was set up in a data-dependent MS/MS mode, with Higher Energy Collision Dissociation (HCD) as the fragmentation method, as described previously^54^.

Microbial peptide identification was carried out using the Proteome Discoverer informatic platform (version 2.0; Thermo Fisher Scientific), with Sequest-HT as search engine and Percolator for peptide validation (FDR < 1%). Search parameters were set as described previously^43^. A custom collection of metagenomic sequences obtained in house from rat faecal samples (7,422,716 sequences in total, deposited in PRIDE along with the whole MS data) and processed according to previous reports^55^ was employed as sequence database.

Host peptide identification was carried out using the Proteome Discoverer informatic platform (version 1.4; Thermo Fisher Scientific), using the protein sequences belonging to the order Rodentia and deposited in UniProtKB/SwissProt (release 2017_05; 26,536 sequences in total) as database. Search engine and parameters were as described above for microbial peptide identification.

The mass spectrometry proteomics data have been deposited to the ProteomeXchange Consortium via the PRIDE^56^ partner repository with the dataset identifier PXD007252.

Taxonomic and functional annotation was performed using multiple strategies. MEGAN v.6.6.7 was used as first annotation option^57^. Protein sequences were preliminary subjected to a DIAMOND (v.0.8.22) search against the NCBI-nr database (2016/09 update), using the blastp command with default parameters^58^; then, DIAMOND outputs were loaded on MEGAN and both lowest common ancestor (LCA) classification and functional annotation (according to InterPro and eggNOG modules) were performed using default parameters. Furthermore, the Unipept web application (v.3.1; https://unipept.ugent.be) was used to carry out an LCA classification of the identified peptide sequences^59^. Finally, an additional functional annotation was accomplished by aligning the identified protein sequences against a database containing all bacterial sequences from UniProtKB/Swiss-Prot (release 2016_09) using DIAMOND (blastp module, e-value threshold 10^−5^); UniProtKB/Swiss-Prot accession numbers were subsequently exploited to retrieve protein name and KEGG orthologous group information from the UniProt website via the ‘retrieve’ tool^60^. Taxonomic information from different sources were combined, giving priority to MEGAN results; functional information from different sources were inspected, merged and made uniform manually. Butyrate, propionate and acetate biosynthetic pathways were reconstructed based on the corresponding KEGG pathway maps^61^, available at http://www.genome.jp/kegg/pathway.html.

### DNA extraction and 16S rDNA gene sequencing

DNA was extracted from the 17 faecal samples collected in the second experiment. Extraction was performed according to QIAamp Fast Stool Kit protocol (QIAGEN, Hilden, Germany). The extracted DNA was purified according to E.Z.N.A.® Soil DNA Kit (Omega Bio-Tek, Norcross, GA). DNA quality and yield were evaluated via agarose gel and Qubit fluorometer (Life Technologies, CA, USA). Libraries were constructed using Illumina’s recommendations as implemented in 16S Metagenomic Sequencing Library Preparation guide. To amplify the variable region 4 of the 16S rRNA gene, we used the 515F and 806R primers (GTGCCAGCMGCCGCGGTAA and GGACTACHVGGGTWTCTAAT, respectively) modified to contain adaptors for MiSeq sequencing. Two separate gene amplification reactions were performed for each sample, pooled together and cleaned up using AMPure XP (Beckman Coulter, Brea, CA) magnetic beads. The next PCR attached dual index barcodes and sequencing adapters using the Illumina Nextera XT kit so that the PCR products may be pooled and sequenced directly. A final library size and quantification was conducted using a Bioanalyzer 2100 (Agilent Technologies, Santa Clara, California, USA). DNA sequencing was performed on the Illumina MiSeq platform using v2 chemistry according to the manufacturer’s specifications to generate paired-end reads of 201 bases in length in each direction. Data quality control and analyses were performed using the QIIME pipeline (v.1.9.1)^62^. The overlapping paired-end reads were merged using the script join_paired_ends.py inside the QIIME package. Only Illumina reads with a length >200 bp were retained for further analysis. OTUs generation was done using a pipeline based on USEARCH’s OTU clustering recommendations (http://www.drive5.com/usearch/manual/otu_clustering.html) using the closed-reference OTU picking to allow clustering of 16S sequences, as previously described^63^. Reads were clustered at 97% identity using UCLUST to produce OTUs^64^. Taxonomy was then assigned using the Greengenes 13_8 database^65^.

### Statistical analysis and graph generation

Count data (spectral counts for metaproteomic results and read counts for 16S rDNA gene sequencing results) were uploaded to the web application MicrobiomeAnalyst (http://www.microbiomeanalyst.ca) to assess differential abundance through comparative statistical analysis^66^. Features with prevalence in <10% of samples in a given comparison were filtered out. Count data were transformed prior to statistical testing according to the Relative Log Expression method^67^. Differential abundance analysis was then carried out using the edgeR algorithm^68^, with an adjusted p-value (FDR) cut-off of 0.05.

Relative abundance data (obtained by dividing the count data by the total number of counts in a sample) were employed to perform a Principal Component Analysis and generate PCA plots using the web application ClustVis (http://biit.cs.ut.ee/clustvis) with default parameters^69^. Heatmaps were generated starting from relative abundance data using the web application Morpheus (https://clue.io/morpheus). Relative abundance values were transformed by subtracting the median abundance of a given taxonomic/functional feature in the dataset, and then dividing by the median absolute deviation, according to one of the ‘transform’ options available in the ‘color scheme’ menu. Scatter plots were created using GraphPad Prism (v.5.03) starting from relative abundance data, and Student’s *t* test was applied to calculate significance of differences between means of SCFA biosynthetic enzyme abundances.

## Electronic supplementary material

Supplementary information

Dataset S1

Dataset S2

## Data Availability

Mass spectrometry proteomics data can be accessed via the PRIDE partner repository with the dataset identifier PXD007252.
