# Supplementary material for: Caloric restriction promotes functional changes involving short-chain fatty acid biosynthesis in the rat gut microbiota
Source: Sci Rep. 2018 Oct 3;8:14778. doi: 10.1038/s41598-018-33100-y (PMC6170429; doi:10.1038/s41598-018-33100-y)
Supplement: Supplementary file 1 — Supplementary information [file 41598_2018_33100_MOESM1_ESM.pdf]

Caloric restriction promotes functional changes involving short-chain fatty acid biosynthesis in the rat gut microbiota

Alessandro Tanca[1], Marcello Abbondio[2], Antonio Palomba[1], Cristina Fraumene[1], Fabio Marongiu[3], Monica Serra[3], Daniela Pagnozzi[1], Ezio Laconi[3], Sergio Uzzau[1,2*]

[1]Porto Conte Ricerche, Science and Technology Park of Sardinia, Tramariglio, Alghero, Italy

[2]Department of Biomedical Sciences, University of Sassari, Sassari, Italy

[3]Department of Biomedical Sciences, University of Cagliari, Cagliari, Italy

*Correspondence and requests for materials should be addressed to S.U. (email: uzzau@portocontericerche.it)

Supplementary information

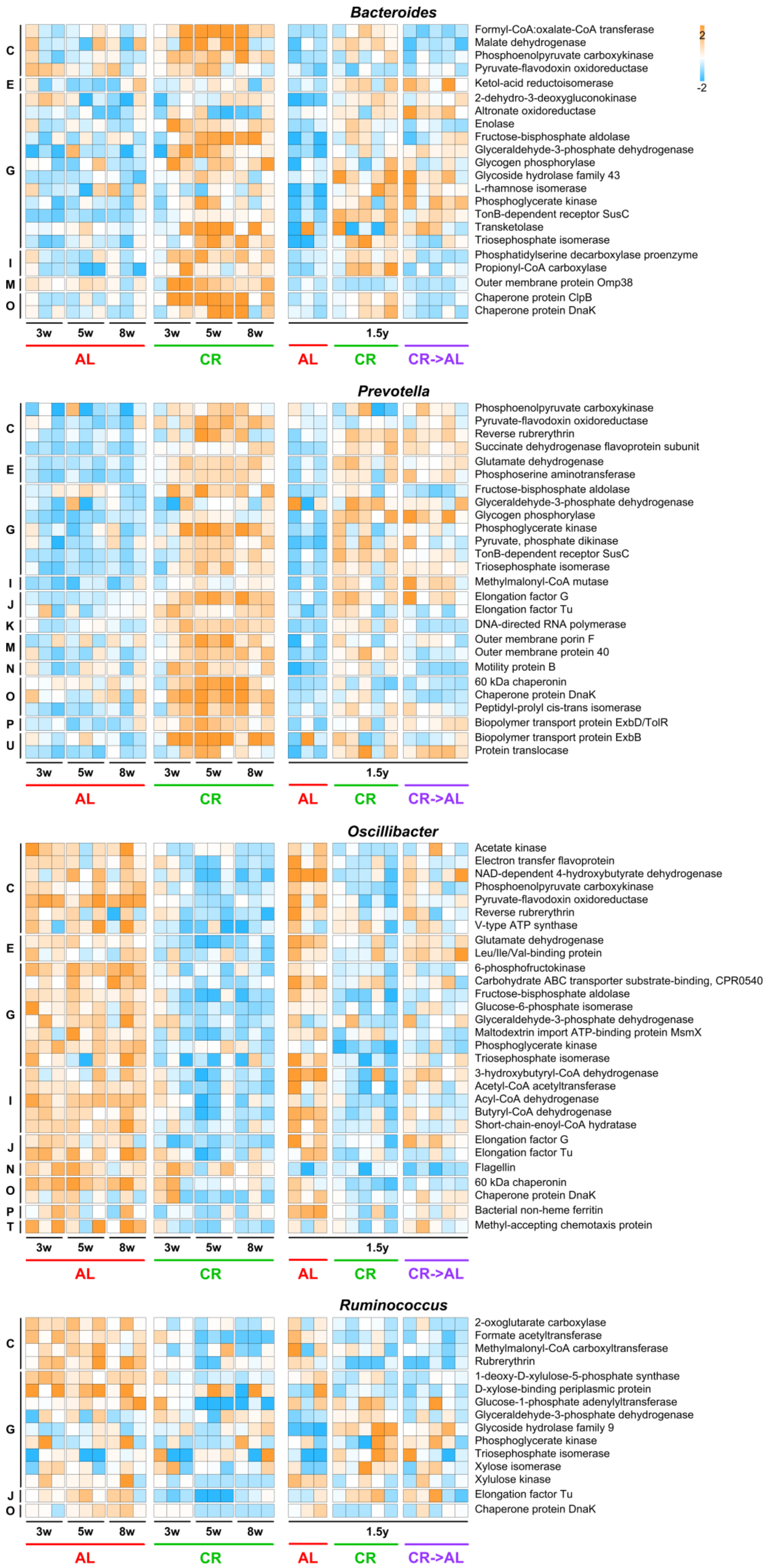
Bacteroides
C
E
G
I
M
O
Formyl-CoA:oxalate-CoA transferase
Malate dehydrogenase
Phosphoenolpyruvate carboxykinase
Pyruvate-flavodoxin oxidoreductase
Ketol-acid reductoisomerase
2-dehydro-3-deoxygluconokinase
Altronate oxidoreductase
Enolase
Fructose-bisphosphate aldolase
Glyceraldehyde-3-phosphate dehydrogenase
Glycogen phosphorylase
Glycoside hydrolase family 43
L-rhamnose isomerase
Phosphoglycerate kinase
TonB-dependent receptor SusC
Transketolase
Triosephosphate isomerase
Phosphatidylserine decarboxylase proenzyme
Propionyl-CoA carboxylase
Outer membrane protein Omp38
Chaperone protein ClpB
Chaperone protein DnaK
2
-2
3w 5w 8w 3w 5w 8w 1.5y
AL CR AL CR CR->AL
Prevotella
C
E
G
I
J
K
M
N
O
P
U
Phosphoenolpyruvate carboxykinase
Pyruvate-flavodoxin oxidoreductase
Reverse rubrerythrin
Succinate dehydrogenase flavoprotein subunit
Glutamate dehydrogenase
Phosphoserine aminotransferase
Fructose-bisphosphate aldolase
Glyceraldehyde-3-phosphate dehydrogenase
Glycogen phosphorylase
Phosphoglycerate kinase
Pyruvate, phosphate dikinase
TonB-dependent receptor SusC
Triosephosphate isomerase
Methylmalonyl-CoA mutase
Elongation factor G
Elongation factor Tu
DNA-directed RNA polymerase
Outer membrane porin F
Outer membrane protein 40
Motility protein B
60 kDa chaperonin
Chaperone protein DnaK
Peptidyl-prolyl cis-trans isomerase
Biopolymer transport protein ExbD/TolR
Biopolymer transport protein ExbB
Protein translocase
3w 5w 8w 3w 5w 8w 1.5y
AL CR AL CR CR->AL
Oscillibacter
C
E
G
I
J
N
O
P
T
Acetate kinase
Electron transfer flavoprotein
NAD-dependent 4-hydroxybutyrate dehydrogenase
Phosphoenolpyruvate carboxykinase
Pyruvate-flavodoxin oxidoreductase
Reverse rubrerythrin
V-type ATP synthase
Glutamate dehydrogenase
Leu/Ile/Val-binding protein
6-phosphofructokinase
Carbohydrate ABC transporter substrate-binding, CPR0540
Fructose-bisphosphate aldolase
Glucose-6-phosphate isomerase
Glyceraldehyde-3-phosphate dehydrogenase
Maltodextrin import ATP-binding protein MsmX
Phosphoglycerate kinase
Triosephosphate isomerase
3-hydroxybutyryl-CoA dehydrogenase
Acetyl-CoA acetyltransferase
Acyl-CoA dehydrogenase
Butyryl-CoA dehydrogenase
Short-chain-enoyl-CoA hydratase
Elongation factor G
Elongation factor Tu
Flagellin
60 kDa chaperonin
Chaperone protein DnaK
Bacterial non-heme ferritin
Methyl-accepting chemotaxis protein
3w 5w 8w 3w 5w 8w 1.5y
AL CR AL CR CR->AL
Ruminococcus
C
G
J
O
2-oxoglutarate carboxylase
Formate acetyltransferase
Methylmalonyl-CoA carboxyltransferase
Rubrerythrin
1-deoxy-D-xylulose-5-phosphate synthase
D-xylose-binding periplasmic protein
Glucose-1-phosphate adenylyltransferase
Glyceraldehyde-3-phosphate dehydrogenase
Glycoside hydrolase family 9
Phosphoglycerate kinase
Triosephosphate isomerase
Xylose isomerase
Xylulose kinase
Elongation factor Tu
Chaperone protein DnaK
3w 5w 8w 3w 5w 8w 1.5y
AL CR AL CR CR->AL

Figure S1. Functional expression profile of *Bacteroides*, *Prevotella*, *Oscillibacter*, and *Ruminococcus* metaproteomes. Relative abundance values concerning the young rat experiment (left, up to 8 weeks of treatment) and adult rat experiment (right, 1.5 years of treatment) are shown. AL, *ad libitum*; CR, caloric restriction; CR→AL, 1-week reversion from caloric restriction to *ad libitum*. Heatmap columns represent samples, while rows represent functions. Only functions with abundance > 0.25% (*Prevotella*) >0.1% (*Bacteroides* and *Oscillibacter*) and >0.05% (*Ruminococcus*) are shown (ribosomal proteins were excluded), and ordered according to the COG category to which they belong.

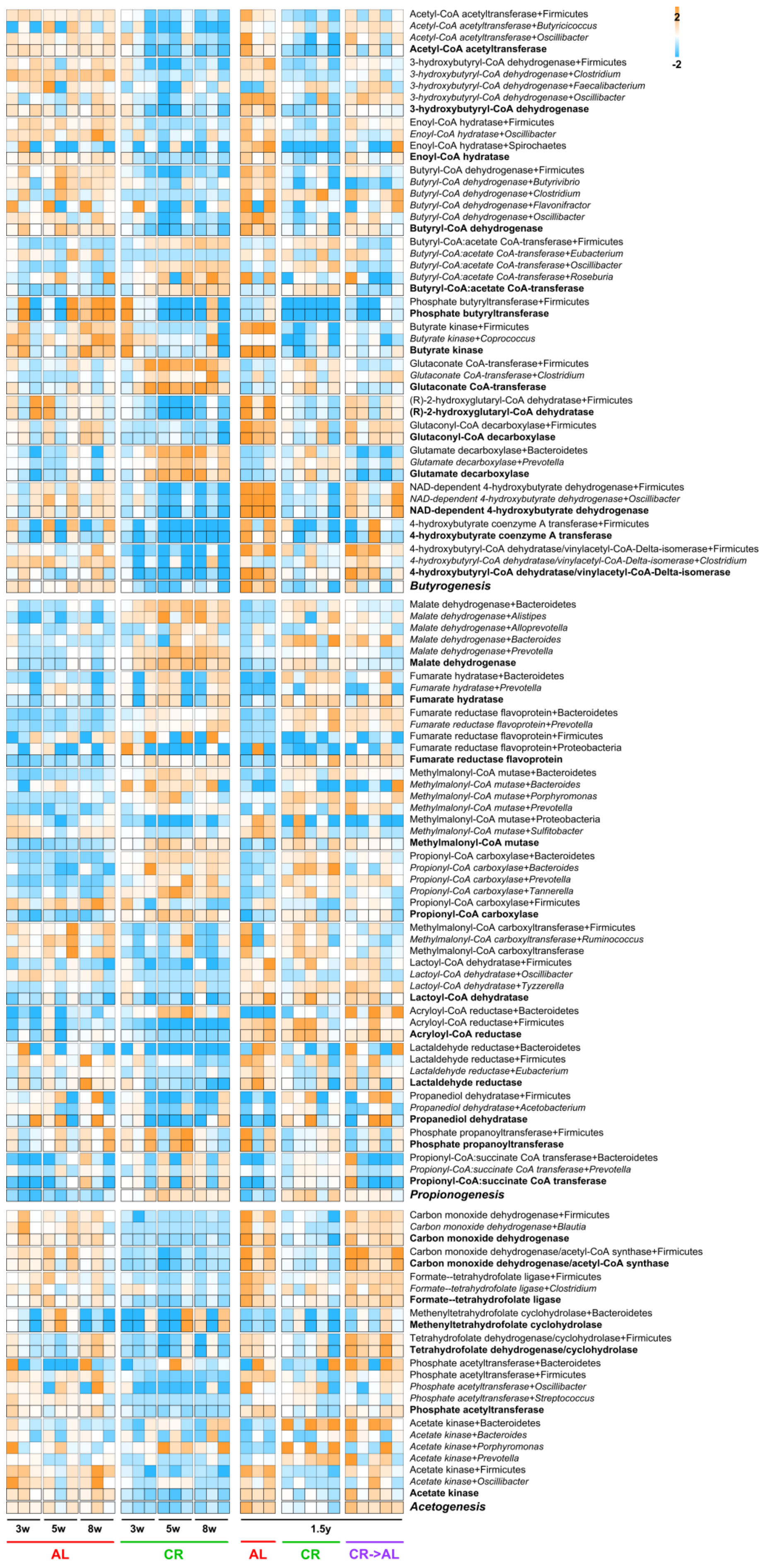
2
-2
Acetyl-CoA acetyltransferase+Firmicutes
Acetyl-CoA acetyltransferase+Butyricicoccus
Acetyl-CoA acetyltransferase+Oscillibacter
Acetyl-CoA acetyltransferase
3-hydroxybutyryl-CoA dehydrogenase+Firmicutes
3-hydroxybutyryl-CoA dehydrogenase+Clostridium
3-hydroxybutyryl-CoA dehydrogenase+Faecalibacterium
3-hydroxybutyryl-CoA dehydrogenase+Oscillibacter
3-hydroxybutyryl-CoA dehydrogenase
Enoyl-CoA hydratase+Firmicutes
Enoyl-CoA hydratase+Oscillibacter
Enoyl-CoA hydratase+Spirochaetes
Enoyl-CoA hydratase
Butyryl-CoA dehydrogenase+Firmicutes
Butyryl-CoA dehydrogenase+Butyrivibrio
Butyryl-CoA dehydrogenase+Clostridium
Butyryl-CoA dehydrogenase+Flavonifractor
Butyryl-CoA dehydrogenase+Oscillibacter
Butyryl-CoA dehydrogenase
Butyryl-CoA:acetate CoA-transferase+Firmicutes
Butyryl-CoA:acetate CoA-transferase+Eubacterium
Butyryl-CoA:acetate CoA-transferase+Oscillibacter
Butyryl-CoA:acetate CoA-transferase+Roseburia
Butyryl-CoA:acetate CoA-transferase
Phosphate butyryltransferase+Firmicutes
Phosphate butyryltransferase
Butyrate kinase+Firmicutes
Butyrate kinase+Coprococcus
Butyrate kinase
Glutaconate CoA-transferase+Firmicutes
Glutaconate CoA-transferase+Clostridium
Glutaconate CoA-transferase
(R)-2-hydroxyglutaryl-CoA dehydratase+Firmicutes
(R)-2-hydroxyglutaryl-CoA dehydratase
Glutaconyl-CoA decarboxylase+Firmicutes
Glutaconyl-CoA decarboxylase
Glutamate decarboxylase+Bacteroidetes
Glutamate decarboxylase+Prevotella
Glutamate decarboxylase
NAD-dependent 4-hydroxybutyrate dehydrogenase+Firmicutes
NAD-dependent 4-hydroxybutyrate dehydrogenase+Oscillibacter
NAD-dependent 4-hydroxybutyrate dehydrogenase
4-hydroxybutyrate coenzyme A transferase+Firmicutes
4-hydroxybutyrate coenzyme A transferase
4-hydroxybutyryl-CoA dehydratase/vinylacetyl-CoA-Delta-isomerase+Firmicutes
4-hydroxybutyryl-CoA dehydratase/vinylacetyl-CoA-Delta-isomerase+Clostridium
4-hydroxybutyryl-CoA dehydratase/vinylacetyl-CoA-Delta-isomerase
Butyrogenesis
Malate dehydrogenase+Bacteroidetes
Malate dehydrogenase+Alistipes
Malate dehydrogenase+Alloprevotella
Malate dehydrogenase+Bacteroides
Malate dehydrogenase+Prevotella
Malate dehydrogenase
Fumarate hydratase+Bacteroidetes
Fumarate hydratase+Prevotella
Fumarate hydratase
Fumarate reductase flavoprotein+Bacteroidetes
Fumarate reductase flavoprotein+Prevotella
Fumarate reductase flavoprotein+Firmicutes
Fumarate reductase flavoprotein+Proteobacteria
Fumarate reductase flavoprotein
Methylmalonyl-CoA mutase+Bacteroidetes
Methylmalonyl-CoA mutase+Bacteroides
Methylmalonyl-CoA mutase+Porphyromonas
Methylmalonyl-CoA mutase+Prevotella
Methylmalonyl-CoA mutase+Proteobacteria
Methylmalonyl-CoA mutase+Sulfitobacter
Methylmalonyl-CoA mutase
Propionyl-CoA carboxylase+Bacteroidetes
Propionyl-CoA carboxylase+Bacteroides
Propionyl-CoA carboxylase+Prevotella
Propionyl-CoA carboxylase+Tannerella
Propionyl-CoA carboxylase+Firmicutes
Propionyl-CoA carboxylase
Methylmalonyl-CoA carboxyltransferase+Firmicutes
Methylmalonyl-CoA carboxyltransferase+Ruminococcus
Methylmalonyl-CoA carboxyltransferase
Lactoyl-CoA dehydratase+Firmicutes
Lactoyl-CoA dehydratase+Oscillibacter
Lactoyl-CoA dehydratase+Tyzzerella
Lactoyl-CoA dehydratase
Acryloyl-CoA reductase+Bacteroidetes
Acryloyl-CoA reductase+Firmicutes
Acryloyl-CoA reductase
Lactaldehyde reductase+Bacteroidetes
Lactaldehyde reductase+Firmicutes
Lactaldehyde reductase+Eubacterium
Lactaldehyde reductase
Propanediol dehydratase+Firmicutes
Propanediol dehydratase+Acetobacterium
Propanediol dehydratase
Phosphate propanoyltransferase+Firmicutes
Phosphate propanoyltransferase
Propionyl-CoA:succinate CoA transferase+Bacteroidetes
Propionyl-CoA:succinate CoA transferase+Prevotella
Propionyl-CoA:succinate CoA transferase
Propionogenesis
Carbon monoxide dehydrogenase+Firmicutes
Carbon monoxide dehydrogenase+Blautia
Carbon monoxide dehydrogenase
Carbon monoxide dehydrogenase/acetyl-CoA synthase+Firmicutes
Carbon monoxide dehydrogenase/acetyl-CoA synthase
Formate--tetrahydrofolate ligase+Firmicutes
Formate--tetrahydrofolate ligase+Clostridium
Formate--tetrahydrofolate ligase
Methenyltetrahydrofolate cyclohydrolase+Bacteroidetes
Methenyltetrahydrofolate cyclohydrolase
Tetrahydrofolate dehydrogenase/cyclohydrolase+Firmicutes
Tetrahydrofolate dehydrogenase/cyclohydrolase
Phosphate acetyltransferase+Bacteroidetes
Phosphate acetyltransferase+Firmicutes
Phosphate acetyltransferase+Oscillibacter
Phosphate acetyltransferase+Streptococcus
Phosphate acetyltransferase
Acetate kinase+Bacteroidetes
Acetate kinase+Bacteroides
Acetate kinase+Porphyromonas
Acetate kinase+Prevotella
Acetate kinase+Firmicutes
Acetate kinase+Oscillibacter
Acetate kinase
Acetogenesis
3w
5w
8w
3w
5w
8w
1.5y
AL
CR
AL
CR
CR->AL

Figure S2. Expression profile of short-chain fatty acid biosynthetic enzymes. Relative abundance values concerning the young rat experiment (left, up to 8 weeks of treatment) and adult rat experiment (right, 1.5 years of treatment) are shown. AL, *ad libitum*; CR, caloric restriction; CR→AL, 1-week reversion from caloric restriction to *ad libitum*. Heatmap columns represent samples, while rows represent functions. Only functions and function-taxon combinations detected in at least half of AL or CR samples are shown. Functions/pathways in bold account for the total abundance of that function/pathway in the metaproteome, independently of the specific taxon to which the function/pathway was assigned.

Table S1. Host proteins with differential abundance between CR and AL fed young rats.

UniProt Accession Number	Host protein	log2fold-change (CR/AL)	FDR
Q6IG00	Keratin, type II cytoskeletal 4	2.55	0.00026769
Q6IFV3	Keratin, type I cytoskeletal 15	1.78	0.02177200
P00687	Alpha-amylase 1	1.75	0.00026769
Q6IMF3	Keratin, type II cytoskeletal 1	1.73	0.02252900
P07882	Bile salt-activated lipase	1.53	0.00327100
Q64319	Neutral and basic amino acid transport protein rBAT	1.46	0.00044596
Q99M73	Keratin, type II cuticular Hb4	1.45	0.02177200
Q02401	Lactase-phlorizin hydrolase	1.44	0.01204500
P01026	Complement C3	1.39	0.00004499
Q6IFV4	Keratin, type I cytoskeletal 13	1.34	0.00055587
Q91WP6	Serine protease inhibitor A3N	1.29	0.00026769
P13676	Acylamino-acid-releasing enzyme	1.24	0.00327930
Q91XT9	Neutral ceramidase	1.18	0.00327100
P05544	Serine protease inhibitor A3L	0.95	0.00052297
P62898	Cytochrome c, somatic	-1.12	0.01714600

Table S2. Host proteins with differential abundance between CR and AL fed old rats.

UniProt Accession Number	Host protein	log2fold-change (CR/AL)	FDR
P54316	Inactive pancreatic lipase-related protein 1	2.93	0.00000032
Q497I4	Keratin, type I cuticular Ha5	2.14	0.00147150
Q99M74	Keratin, type II cuticular Hb2	1.93	0.00913720
Q6IG02	Keratin, type II cytoskeletal 2 epidermal	1.93	0.01366600
Q9Z2T6	Keratin, type II cuticular Hb5	1.46	0.00181370
Q61765	Keratin, type I cuticular Ha1	1.43	0.02710800
Q99M73	Keratin, type II cuticular Hb4	1.38	0.00697830
P07882	Bile salt-activated lipase	1.36	0.00000159
Q6IFW6	Keratin, type I cytoskeletal 10	1.22	0.00913720
Q99MA2	Xaa-Pro aminopeptidase 2	1.19	0.00147150
Q6IMF3	Keratin, type II cytoskeletal 1	1.11	0.02472500
Q9ERE2	Keratin, type II cuticular Hb1	1.06	0.00181370
P23739	Sucrase-isomaltase, intestinal	1.03	0.00190030
Q9D646	Keratin, type I cuticular Ha4	0.98	0.02402500
Q91XT9	Neutral ceramidase	0.87	0.01369700
Q9QZT0	CUB and zona pellucida-like domain-containing protein 1	0.79	0.01051300
P48975	Actin, cytoplasmic 1	-0.73	0.01366600
Q62635	Mucin-2	-0.81	0.00397500
Q9D7Z6	Calcium-activated chloride channel regulator 1	-0.90	0.04141600
Q03626	Murinoglobulin-1	-2.64	0.00125480

Dataset S1. Tables reporting general metrics, identifications, taxonomic and functional annotations and differential features from metaproteomic data.

Dataset S2. Tables reporting taxonomic results from 16S rDNA gene sequencing data.
